# Supplementary material for: A Critical View of the Application of the APEX Software (Aqueous Photochemistry of Environmentally-Occurring Xenobiotics) to Predict Photoreaction Kinetics in Surface Freshwaters
Source: Molecules. 2019 Dec 18;25(1):9. doi: 10.3390/molecules25010009 (PMC7017383; doi:10.3390/molecules25010009)
Supplement: Supplementary file 1 [file molecules-25-00009-s001.zip › 3.2.4_gcc-4.4.0.zip!/3.2.4_gcc-4.4.0/3.2.4_gcc-4.4.0/doc/octave/html/interpreter/The-_003ccode_003ewhile_003c_002fcode_003e-Statement.html]

The @code{while} Statement - Untitled


Next: The <code>do-until</code> Statement,
Previous: The <code>switch</code> Statement,
Up: Statements


---

### 10.3 The `while` Statement

In programming, a loop means a part of a program that is (or at least can
be) executed two or more times in succession.

The `while` statement is the simplest looping statement in Octave.
It repeatedly executes a statement as long as a condition is true. As
with the condition in an `if` statement, the condition in a
`while` statement is considered true if its value is non-zero, and
false if its value is zero. If the value of the conditional expression
in a `while` statement is a vector or a matrix, it is considered
true only if it is non-empty and *all* of the elements are non-zero.

Octave's `while` statement looks like this:

```
     while (condition)
       body
     endwhile
```

Here body is a statement or list of statements that we call the
body of the loop, and condition is an expression that
controls how long the loop keeps running.

The first thing the `while` statement does is test condition.
If condition is true, it executes the statement body. After
body has been executed, condition is tested again, and if it
is still true, body is executed again. This process repeats until
condition is no longer true. If condition is initially
false, the body of the loop is never executed.

This example creates a variable `fib` that contains the first ten
elements of the Fibonacci sequence.

```
     fib = ones (1, 10);
     i = 3;
     while (i <= 10)
       fib (i) = fib (i-1) + fib (i-2);
       i++;
     endwhile
```

Here the body of the loop contains two statements.

The loop works like this: first, the value of `i` is set to 3.
Then, the `while` tests whether `i` is less than or equal to
10. This is the case when `i` equals 3, so the value of the
`i`-th element of `fib` is set to the sum of the previous two
values in the sequence. Then the `i++` increments the value of
`i` and the loop repeats. The loop terminates when `i`
reaches 11.

A newline is not required between the condition and the
body; but using one makes the program clearer unless the body is very
simple.
